# Scaling and universality in glass transition

**DOI:** 10.1038/srep26481

**Published:** 2016-05-25

**Authors:** Antonio de Candia, Annalisa Fierro, Antonio Coniglio

**Affiliations:** 1 Dipartimento di Fisica “Ettore Pancini”, Università di Napoli “Federico II”, Complesso Universitario di Monte Sant’Angelo, via Cintia, 80126 Napoli, Italy; 2 CNR-SPIN, via Cintia, 80126 Napoli, Italy; 3 INFN, Sezione di Napoli, via Cintia, 80126 Napoli, Italy

## Abstract

Kinetic facilitated models and the Mode Coupling Theory (MCT) model B are within those systems known to exhibit a discontinuous dynamical transition with a two step relaxation. We consider a general scaling approach, within mean field theory, for such systems by considering the behavior of the density correlator 〈*q*(*t*)〉 and the dynamical susceptibility 〈*q*^2^(*t*)〉 − 〈*q*(*t*)〉^2^. Focusing on the Fredrickson and Andersen (FA) facilitated spin model on the Bethe lattice, we extend a cluster approach that was previously developed for continuous glass transitions by Arenzon *et al.* (Phys. Rev. E 90, 020301(R) (2014)) to describe the decay to the plateau, and consider a damage spreading mechanism to describe the departure from the plateau. We predict scaling laws, which relate dynamical exponents to the static exponents of mean field bootstrap percolation. The dynamical behavior and the scaling laws for both density correlator and dynamical susceptibility coincide with those predicted by MCT. These results explain the origin of scaling laws and the universal behavior associated with the glass transition in mean field, which is characterized by the divergence of the static length of the bootstrap percolation model with an upper critical dimension *d*_*c*_ = 8.

Many physical systems and models exhibit a sudden slowing down of their dynamics, followed by a dynamical transition associated with a structural arrest. Roughly, we can distinguish two type of transitions, continuous and discontinuous, depending whether or not there is a jump at the threshold of the dynamical correlator in the infinite time limit. An example of the first category is given by the sol-gel transition (e.g. refs 1, 2, 3). This dynamical transition was studied using a cluster approach, based on percolation theory^4^. An explicit scaling form for the dynamical correlator was found, and general scaling laws connecting the dynamical exponents with the random percolation exponents were derived. Recently, it was shown^5^,^6^ that using mean field percolation exponents, the same scaling form for the correlator and the same scaling relations were also valid for the continuous transition of MCT model A^7^, suggesting that the origin of the continuous MCT scaling relations is due to an underlying static transition in the same universality class of random percolation. In the MCT schematic model, the correlator *ϕ*(*t*) obeys the integro-differential equation: 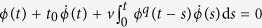, where *q* = 1 in the continuous model A and *q* = 2 in the discontinuous model B, *v* is the controlling parameter and *t*_0_ is a characteristic microscopic timescale^7^.

The glass transition instead belongs to the second category, characterized by discontinuous transition. A great advance in glass theory was provided by MCT developed by Götze and collaborators^7^,^8^,^9^. This theory starting from first principles, under some mean field approximations, predicts a dynamical arrest at a finite temperature *T*_*c*_, characterized by power law behavior and universal scaling laws. These theoretical predictions have been tested in great detail both experimentally and numerically^10^,^11^,^12^,^13^,^14^,^15^. A schematic version is given by the MCT discontinuous model B, introduced above. Other models, like *p*-spin glass models (e.g. refs 16,17), Random Field Ising model in an external field (e.g. refs 18, 19, 20, 21), kinetic facilitated models (e.g. refs 22, 23, 24), reproduce in mean field the same dynamical behavior and scaling laws. However the transition described by MCT does not seem to exhibit any critical change in the structure and no diverging static length. How can we then explain the scaling laws and universal behavior found at mean field level? In this paper we consider as paradigmatic example the Fredrickson and Andersen facilitated Ising model^22^ on a Bethe lattice^25^. The infinite time limit of the persistence of this model, Φ(*t*), tends^25^ to the order parameter of the bootstrap percolation (BP) model^26^,^27^,^28^. The BP model exhibits a mixed order transition with an order parameter which jumps discontinuously at the transition, nevertheless the fluctuations, and the critical length associated to it, diverge as the transition is approached from the glassy phase.

Generalizing the cluster approach considered for the continuous dynamic transition^5^,^6^, we are able to predict the dynamical behavior for the correlator and for the dynamical susceptibility of the FA facilitated model, including universal scaling laws that relate dynamical exponents with the static universal exponents of BP. Using the mean field values of these static exponents, we find that the dynamical behavior and the scaling laws are the same as predicted by MCT model B, what validates the early suggestion^25^,^29^,^30^,^31^,^32^ that the facilitated model and the MCT model B have a similar dynamical behavior. Akin the results found for the continuous transition^5^,^6^, using the cluster approach we find a new more precise form for the approach of the correlator to the plateau, characterized by a power law, followed by a stretched exponential divided by a power law. These new predictions are verified numerically on both FA facilitated model and MCT model B. All these results suggest a general common mechanism for discontinuous glass transition at mean field level, based on a static transition in the same universality class of bootstrap percolation with a diverging static length, which is responsible for the origin of scaling and universality present in such a wide range of systems, apparently very different from each other.

Here, for convenience, we summarize the main results. Given a two step relaxation, the correlator can be written as 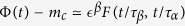, where *m*_*c*_ is the value of the plateau at transition, *β* = 1/2 is the order parameter BP exponent, 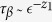 corresponds to the first step relaxation to the plateau, and 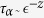 corresponds to the second step relaxation time. At criticality Φ(*t*) − *m*_*c*_ ~ *t*^−*a*^, with *z*_1_ = *β*/*a* = 1/2*a*, while the approach to the plateau is given by a stretched exponential divided by a power law with precise predictions following from the cluster approach. The departure from the plateau is given by Φ(*t*) − *m*_*c*_ ~ −*ϵ*^*β*^(*t*/*τ*_*β*_)^*b*^, which is interpreted as damage propagating from an initial density of infected sites *ϵ*^*β*^, times (*t*/*τ*_*β*_)^*b*^, the number of distinct damaged sites by one initial infected site during the time *t*. A consequence of the scaling function of the two variables is the scaling relation between dynamic exponents *a*, *b*, *z* and the BP static exponent *β*, *z* = *β*/*a* + *β*/*b* = 1/2*a* + 1/2*b*. The dynamical susceptibility, 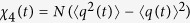 (where *N* is the number of particles) in the liquid phase is given by 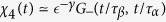, where *γ* = 1 is the BP critical exponent of the fluctuation of the order parameter. This scaling leads to *χ*_4_(*t*) ~ *t*^*aγ*/*β*^ = *t*^2*a*^ for *t* < *τ*_*β*_ with a crossover to *t*^2*b*^ for 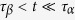. This crossover is a consequence that the dynamics in this regime is due to propagation of damage and that *χ*_4_(*t*) is proportional to the square of distinct damaged sites. Finally, *χ*_4_(*t* = *τ*_*β*_) ~ *ϵ*^−*γ*^ = *ϵ*^−1^ and *χ*_4_(*t* = *τ*_*α*_) ~ *ϵ*^−*γ*−2*β*^ = *ϵ*^−2^ and goes to zero in the infinite time limit. In the glassy phase *χ*_4_(*t*) ~ *t*^*aγ*/*β*^ = *t*^2*a*^ for *t* < *τ*_*β*_ with a crossover to a constant plateau whose value diverges as ~*ϵ*^−*γ*^ = *ϵ*^−1^.

In the following, using the cluster approach and a damage spreading mechanism, we will derive on the Bethe lattice the dynamical behavior of the correlator and the dynamical susceptibility in terms of critical exponents of the BP model, and compare with MCT results. In the supplementary information, we calculate the critical exponents of the BP model, where in particular it is stressed the difference between the behavior of the mean cluster size of the corona clusters, which diverges with an exponent *γ*′ = 1/2, and the fluctuation of the percolation order parameter, which diverges with an exponent *γ* = 1.

## Results

### Kinetic facilitated models and bootstrap percolation

**Figure 1:**
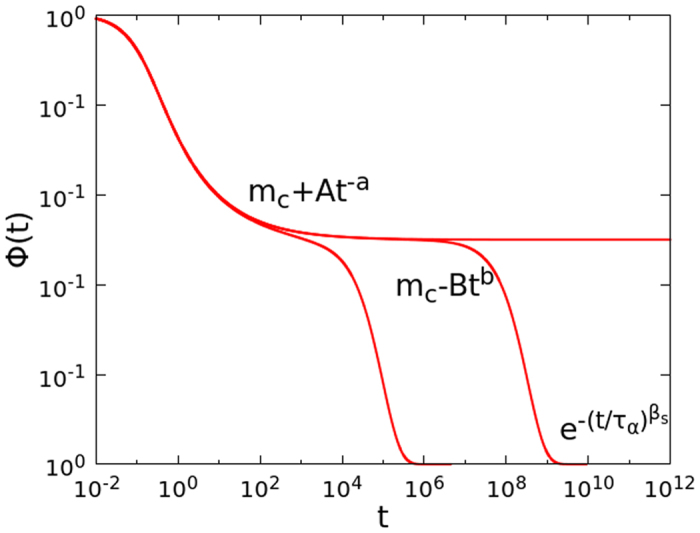
Correlators in MCT schematic model B. Each curve corresponds to a different value of *ϵ*. The correlator reaches the plateau with a power law decay, Φ(*t*) − *m*_*c*_ ~ *t*^−*a*^, and the departure from the plateau is given by Φ(*t*) − *m*_*c*_ ~ −(*t*/*τ*_*β*_)^*b*^. At long times, a crossover is observed to a new regime, well fitted by stretched exponential function.

Kinetic facilitated models^24^ like Fredrickson and Andersen^22^ or Kob and Andersen models^23^ on the Bethe lattice have been suggested^25^ to have a discontinuous MCT-like transition (see Fig. 1)^7^,^8^,^9^. Our objective is to use a physical picture to understand the origin and the mechanism leading to such peculiar dynamical behavior. In order to do so, we consider, in particular, the Fredrickson and Andersen (FA) kinetic facilitated model^22^ (FA) on a Bethe lattice. The FA model is defined on a lattice, where an Ising variable, *S*_*i*_ = ±1, is assigned to each of the *N* sites, with Hamiltonian, 
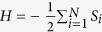
. The spins variables are updated according to the standard spin flip dynamics, along with the constraint that a spin can only flip if the number of nearest neighbors in the down state (*S*_*i*_ = −1) is larger than or equal to *f*.

The dynamics of the system can be characterized by the correlator, Φ(*t*) = 〈*q*(*t*)〉, a quantitative measure of how the system configuration at time *t* is correlated to the configuration at time *t* = 0, the dynamical susceptibility, 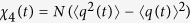, and the dynamical pair correlation function, *g*_*ij*_(*t*), where


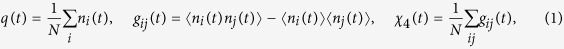


with *n*_*i*_(*t*) = 0, 1 depending whether a spin at site *i* has flipped or not during time interval (0, *t*), respectively^33^.

On a Bethe lattice of coordination number *z* = *k* + 1, the model, for 0 < *f* < *k* − 1, has a transition from a liquid phase at high temperatures (where the density of down spins is large), to a frozen phase at low temperatures, where down spins are few and an infinite cluster of blocked spins appears^34^,^35^,^36^. It was shown^25^ that in the *t* → ∞ limit, this transition corresponds exactly to that of BP. Bootstrap percolation has a mixed order transition: while the percolation order parameter *P* of BP jumps discontinuously at the threshold from zero to *P*_*c*_, the fluctuation *χ* of the order parameter with respect to the initial configuration, and the associated length *ξ* diverge according to:


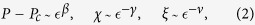


where


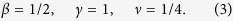


The behavior of the order parameter characterized by the exponent *β* = 1/2 was first derived in the original paper where BP was first introduced^26^, Note that the fluctuation of the order parameter *χ* must not be confused with the mean cluster size of the “corona”. These are clusters made of sites belonging to the percolating cluster, surrounded by a number of facilitated sites exactly equal to *f* − 1^27^. The mean cluster size of the corona in fact diverges with an exponent 1/2^27^,^28^. In ref. 27 a second correlation function was introduced leading to a second “susceptibility” diverging with an exponent 1, but it is not clear whether this quantity is related to the fluctuation of the percolation order parameter. In the Supplementary Information, we calculate explicitly the fluctuation of the order parameter and show that it diverges with an exponent *γ* = 1 along with the associated correlation length *v* = 1/4.

In summary, for the FA model we have, in the glassy phase, *m* = *P*, *χ*_4_(∞) = *χ*, and *ξ*_4_(∞) = *ξ*, where *m* = lim_*t*→∞_ Φ(*t*), 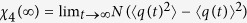 are the FA order parameter and its fluctuation, respectively, and *ξ*_4_(∞) is the associated length.

#### Decay to the plateau using the cluster approach

We note that coming from the glassy phase *T* < *T*_*c*_, the static properties of the FA model exhibits a mixed order transition at the critical temperature *T*_*c*_, whose critical behavior is given by Eq. (2). However, by re-defining the order parameter as *m* − *m*_*c*_, the transition can be considered as a continuous one. Therefore, we can apply the cluster formalism developed for the continuous transition, such as the sol-gel transition and the dynamical transition of the MCT model A^4^,^5^,^6^.

In the cluster approach, it is assumed that the system can be described by a distribution of clusters *n*(*s*), where each cluster of size *s* decays with a simple exponential


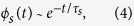


where *τ*_*s*_ is the relaxation time of a cluster of size *s*. The larger the size of the cluster, the larger is the relaxation time. It is natural to assume the following power law relation, as usually found for polymer systems:


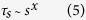


where *x* is a constant exponent. The density correlator of the entire system is given by the sum over all clusters


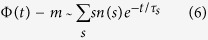


where


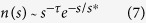


is the cluster distribution associated to the fluctuation of BP with *τ* = 2 + *β*/(*β* + *γ*) and s* = *ϵ*^−(*β*+*γ*)^, where *β* = 1/2 and *γ* = 1 are the mean field BP exponents. In the sol-gel transition and MCT model A, the cluster distribution is given by random percolation theory with *β* = 1 and *γ* = 1^37^. Note that this approach is rather general, it is based only under the assumption that the system configuration can be partitioned in a distribution of clusters, each decaying with a relaxation time proportional to *s*^*x*^. Even if we do not know precisely the cluster definition, the approach is still valid, just like in a liquid-gas transition close to the critical point it is appropriate to describe the critical properties in terms of a distribution of droplets, in the spirit of Fisher’s droplet model^38^,^39^,^40^.

Provided that we are in the glassy phase, *T* ≤ *T*_*c*_, we can apply the cluster formalism of the continuous transition, which predicts a pure power law decay^4^,^5^,^6^ for the entire range of times at the transition, *T* = *T*_*c*_, and the same power law below *T*_*c*_, provided that 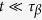,


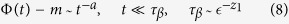



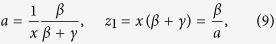


where *x* is related to the relaxation time of a fluctuation of size *s* by Eq. (5). Inserting BP mean field exponents *β* = 1/2 and *γ* = 1, we obtain


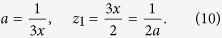


**Figure 2:**
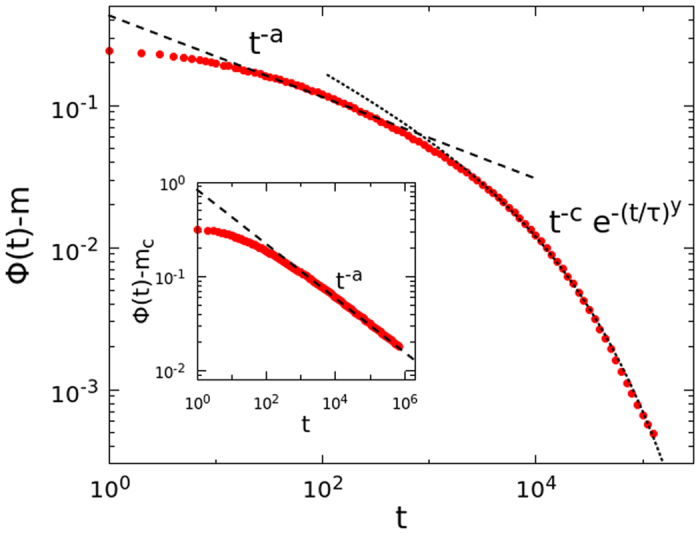
The correlator of the FA model on the Bethe lattice in the glassy phase at *T*_*c*_ − *T* = 2^−7^ (main frame) and at the transition (inset). The dashed line is the power law *t*^−*a*^ with exponent *a* = 0.29. The dotted line in the glassy phase is given by Eq. (11), with *y* = 0.46 and *c* = 0.39.

Moreover, as in the continuous case, close to *T*_*c*_ the power law is followed by a transient, whose behavior is given by a stretched exponential combined with a power law^4^,^5^,^6^:


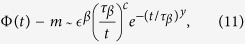


with


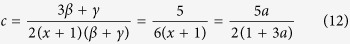


and


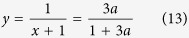



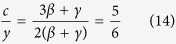


we have performed large scale numerical simulations of the FA model on the Bethe lattice with *k* = 3, *f* = 2 and *N* = 2^18^. Figure 2 shows the correlator in the glassy phase (main frame) and at the transition (inset). The value of *a* ≈ 0.29 is obtained from the power law decay at the critical temperature. From this value, using Eqs (12) and (13), we predict the exponents *y* ≈ 0.46 and *c* ≈ 0.39, defined in Eq. (11). Figure 2 shows that the data are in excellent agreement with the cluster approach predictions.

In the liquid phase, we have the same approach to the plateau, Eq. (8) replacing *m* by *m*_*c*_, and the same power law, Eq. (9), provided that the system is close enough to *T*_*c*_ and 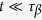. Note that the behavior given by Eq. (11) is much less pronounced in the liquid phase, since for large *t* the regime corresponding to the departure from the plateau will become dominant and interfere with it.

#### Departure from the plateau using the damage spreading mechanism

In the liquid phase, all the clusters (fluctuations) vanish in the long time limit, but they survive on time scales of the order of *τ*_*β*_, when the plateau is still present. The small clusters start to decay first, the last clusters to relax are the largest clusters, i.e. the critical clusters. Once the sites in the critical clusters have moved (relaxed), they act as initial damaged sites to “free” the sites of the potential bootstrap percolating cluster represented by the plateau. As the time increases, the damage spreads through a branching cascade process^41^. The physical picture behind it is that the potential infinite cluster, which contributes to the plateau, is made of a sea of quasi frozen sites, surrounded by critical clusters. Just above the critical temperature the critical clusters eventually decay, whereas just below the critical temperature, the critical clusters themselves become frozen and part of the infinite cluster. The number of sites *m*(*t*) in the core, which are liberated by the damage spreading, is related to the correlator by


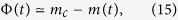



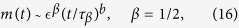


where *ϵ*^*β*^ is the density of sites in the critical clusters and therefore the density of initial damaged sites, and 1/*τ*_*β*_ is, according to the cluster picture, the diffusion coefficient of the sites in the critical cluster, and *b* is a dynamical exponent related to the spreading damage mechanism.

Finally, in the *α* regime,


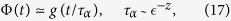


and, like in MCT, using the matching conditions with the previous regime


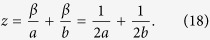


**Figure 3:**
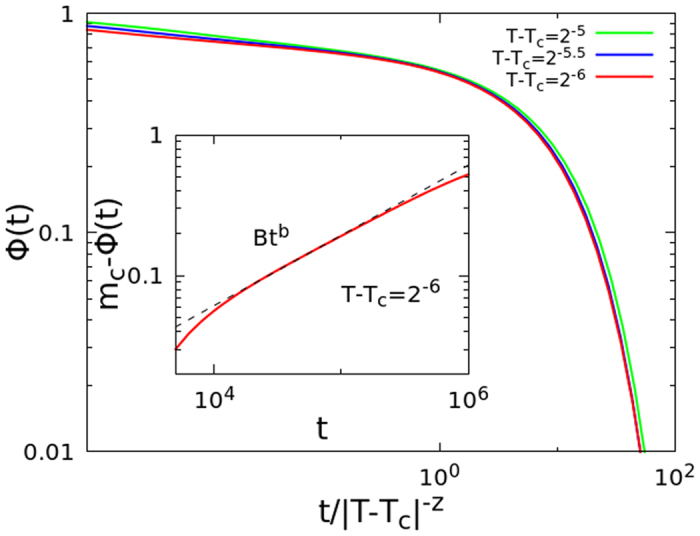
Main frame: Scaling collapse of the correlator of the FA model on the Bethe lattice, in the liquid phase *T* > *T*_*c*_, Eq. (17), with *z* = 2.72. Inset: Departure from the plateau, the dashed line is the power law with *b* = 0.50.

In Fig. 3, we have reported the scaling collapse of the correlator in the *α* regime, Eq. (17), from which the exponent *z* ≈ 2.72 of the relaxation time *τ*_*α*_ has been evaluated. This value is consistent with the value found in ref. 25. In the inset we have also reported the departure from the plateau Eq. (16) and the value of *b* ≈ 0.50 has been evaluated. The exponents *z*, *a* and *b* satisfy not only the scaling relation (16), but *a* and *b* are also found to satisfy the other MCT relation, Eq. (20), with *λ* = 0.785. For convenience, we have reported in Table 1 all the exponents found numerically for the FA model on the Bethe lattice with the estimated errors.

Given two critical times *τ*_*β*_ and *τ*_*α*_, in the liquid phase it may be more convenient to express the density correlator Φ(*t*) as a scaling function of two variables:


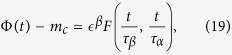


*F*(*x*, *y*) = *F*_1_(*x*) for 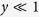, where 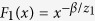 for 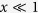 and *F*_1_(*x*) = −*x*^*b*^ for *x* > 1, and *F*(*x*, *y*) = −*x*^*b*^*F*_2_(*y*) for 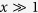 and *y* > 1. The requirement that Φ(*t*) for *t* > *τ*_*α*_ is a func*t*ion of *t*/*τ*_*α*_ only, Eq. (17), implies tha*t ϵ*_*β*_*x*^*b*^ = *y*^*b*^, which in turn leads to ^*τ*^_*α*_ = *τ*^*b*^*τ*_*β*_ where 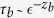 with *z*_*b*_ = 1/2*b*. Taking into account that 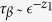, it follows the scaling relation Eq. (18).

### Comparison with discontinuous MCT model B

Interestingly, the correlator of the MCT model B satisfies the same scaling forms Eqs (8), (16) and (17) and scaling relations Eqs (9) and (18), suggesting that the above picture is consistent with MCT. This is further validated if we consider that the mean field static BP exponents coincide with those found in the Random Field Ising (RFI) model in an external field^18^,^19^,^20^,^21^, which was shown to be mapped on the MCT theory, and that both BP and RFI model have an upper critical dimension *d*_*c*_ = 8, which coincides with the value found for MCT^42^.

MCT also predicts a relation between the exponent *a* and *b* and the MCT parameter *λ*:


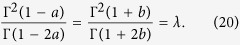


The parameter *λ* for the discontinuous model B is 0.5.

In our approach instead of *λ* we have *x* as parameter, which is related to *a* through


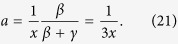


If our approach applies to MCT, *x* can be related to *λ* and consequently to *b* through Eq. (20)


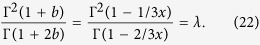


At the moment, we do not have an intuitive physical picture of why *x* and *b* should be related in such a manner. Interestingly, our data show that the MCT relation Eq. (20) is well verified on the FA model, strongly supporting the idea that the FA model in mean field reproduces entirely MCT^29^,^30^,^31^.

**Figure 4:**
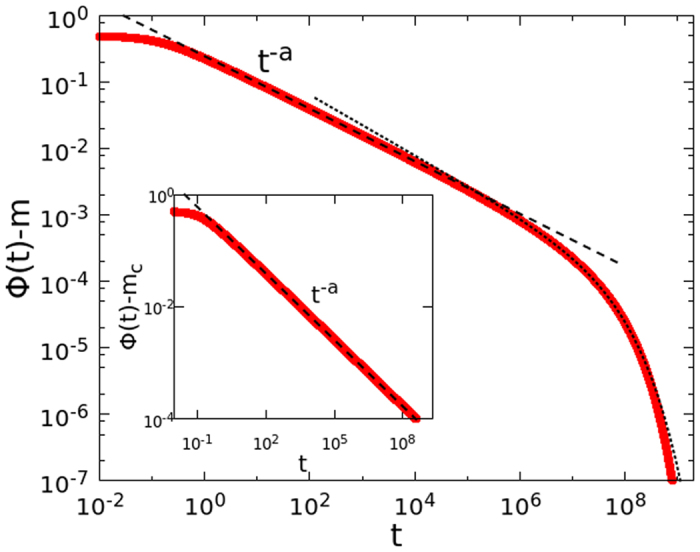
Numerical solution of the MCT schematic model B in the glassy phase (red continuous line), with *λ* = 0.5. Dashed line is the power law *t*^−*a*^, with *a* = 0.40, obtained from Eq. (20). The data are in excellent agreement with the cluster approach prediction (dotted line is the stretched exponential-like decay, Eq. (11), with *c* = 0.45 and *y* = 0.54, obtained using *x* = 1/3*a* = 0.84).

Our approach predicts that the approach to the plateau, after a power law behavior, should be described by the stretched exponential Eq. (11) with exponents given by Eqs (12) and (13), before becoming an exponential decay. By numerically solving the MCT schematic model, we found that the approach to the plateau is very well described by the above predictions (see Fig. 4).

### Fluctuations of the order parameter

Dynamical heterogeneities play an important role in understanding the nature of the glass transition^43^,^44^,^45^,^46^,^47^,^48^,^49^,^50^,^51^,^52^,^53^,^54^,^55^. They are described through the dynamical susceptibility, *χ*_4_(*t*), defined as the fluctuations of the dynamical order parameter: 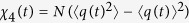. In the following, we will refer to the FA model, however the same predictions apply to the MCT model as well, if the two models behave in the same way, as shown already for the decay of the correlator. As for the correlator, we express *χ*_4_(*t*) as a scaling function of two variables. Since in the glassy phase for t going to infinity *χ*_4_(*t*) coincides with the fluctuation of the BP order parameter, which diverges with an exponent *γ* = 1 as the glass transition is approached we can write:


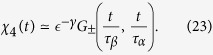


where *G*_±_(*x*, *y*) is a two variables scaling function in the glassy (+) and liquid (−) phase.

#### Glassy phase

Given that in the glassy phase *τ*_*α*_ = ∞, we have


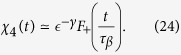


where 
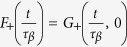
. *F*_+_ = const for *t* → ∞ and 
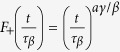
 for *t* < *τ*_*β*_, so that since *τ*_*β*_ ~ *ϵ*^−*β*/*a*^,


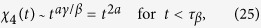


is independent on *ϵ*, and


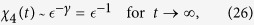


In conclusion in the glassy phase *χ*_4_(*t*) grows as a power law *t*^2*a*^ until it reaches a plateau at *t* ~ *τ*_*β*_, whose value diverges with an exponent *γ* = 1.

#### Liquid phase

**Figure 5:**
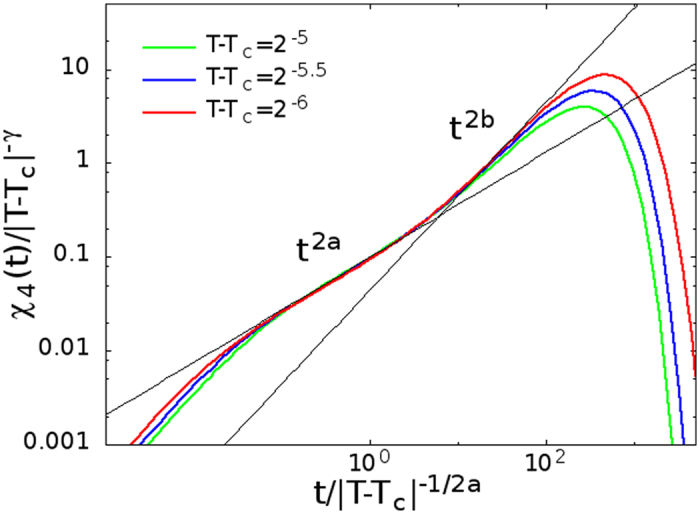
Data collapse in the *β* regime of the dynamical susceptibility, *χ*_4_(*t*), for the FA model on the Bethe lattice, showing the scaling relation Eq. (27) with *γ* = 1, *τ*_*β*_ ~ *ϵ*^−1/2*a*^. Straight lines show the power law behaviors in the early (*t*^2*a*^) and late *β* regime (*t*^2*b*^), with *a* = 0.29 and *b* = 0.50.

In the liquid phase, we have:


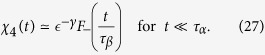


where 
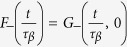
. In the early regime, *t* ≤ *τ*_*β*_, the behavior is the same as in the glassy phase,


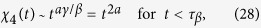



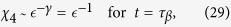


we have numerically verified on FA model on the Bethe lattice the scaling relation Eq. (27). In Fig. 5, we have reported the rescaled susceptibility, showing that in the *β* regime 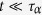 all curves rescale onto a unique function corresponding to *F*_−_(*x*) (with *x* = *t*/*τ*_*β*_), and that *χ*_4_(*t*) ~ *ϵ*^−*γ*^ for *t* = *τ*_*β*_ (with *γ* = 1).

In the late *β* regime, 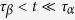, as the dynamical process is due to the damage spreading mechanism, *χ*_4_(*t*) must be proportional to the square of the number of visited sites *m*^2^(*t*) ~ *t*^2*b* ^56 similar to what is found in the diffusing defects mechanism (see refs 33,57), therefore


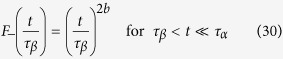


From Eqs (27) and (30)


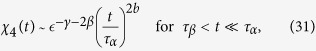


where *τ*_*α*_ ~ *ϵ*^−*z*^, *τ*_*β*_ ~ *ϵ*^−*β*/*a*^ and the scaling relation Eq. (18) has been taken into account.

**Figure 6:**
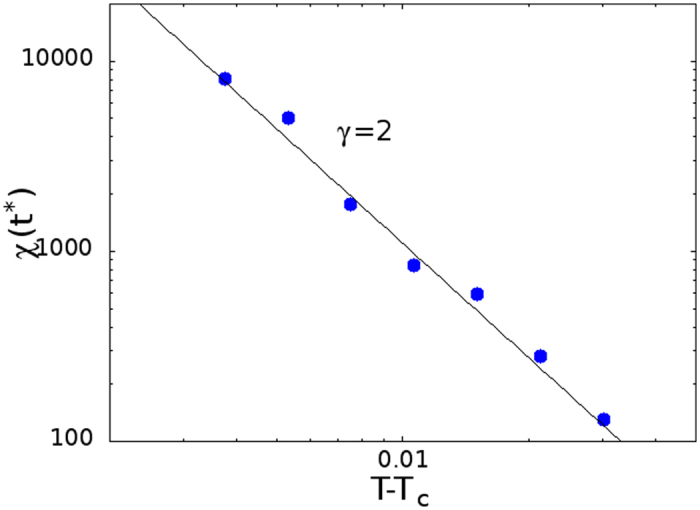
Maximum of the dynamical susceptibility *χ*_4_(t*) for the FA model on the Bethe lattice as a function of *T* − *T*_*c*_. The maximum diverges as |*T* − *T*_*c*_|^2^, in agreement with the prediction of Eq. (33).

In general, in the late *β* and *α* regime *t* > *τ*_*β*_, from Eq. (27) we have 
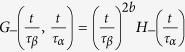
, where *H*_−_(*y*) = const., for *y* ≤ 1 in order to match the behavior in the late *β* regime Eq. (30), and goes to zero for 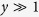, as *χ*_4_(*t*) in the infinite time limit tends to the value of the BP susceptibility *χ*, which is zero in the liquid phase. Therefore


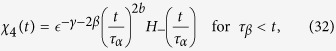



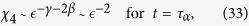


where *β* = 1/2, *γ* = 1, have been taken into account. We have found good agreement for the FA model as shown in Fig. 6, where the maximum of *χ*_4_(*t*) is plotted for *t* = t* ~ *τ*_*α*_, and in Fig. 5, where it is shown *χ*_4_(*t*) ~ *t*^2*a*^ in the early *β* regime and *χ*_4_(*t*) ~ *t*^2*b*^ in the late *β* regime.

*Comparison with MCT-χ*_4_(*t*) was studied within the *p*-spin model by Franz and Parisi^46^ and within the MCT theory by Biroli and Bouchaud^58^ using a diagrammatic approach. The MCT results^57^ predicted for *χ*_4_(*t*) a growth respectively *t*^*a*^ and *t*^*b*^ for the early and late *β* regime and a growth of the maximum at t* with an exponent 1. Later it was argued^59^ that this behavior is valid only for ensembles where all conserved degrees of freedom are fixed, e.g. Newtonian dynamics in the NVE ensemble or Brownian dynamics, in the NVT ensemble, otherwise other diagrams would contribute to *χ*_4_(*t*) leading to a behavior *t*^2*a*^ and *t*^2*b*^ and an exponent 2 for the growth of the maximum of *χ*_4_(*t*) at t*. The same found in our approach. More recently in^60^, this last behavior was found to be much more general, being due to self induced disorder. Changing the initial condition induces fluctuation in the induced disorder leading to the new dynamical behavior.

## Discussion

In conclusion, we have shown that a cluster approach and a damage spreading mechanism, applied to the FA kinetic facilitated model in mean field, predict a discontinuous dynamical transition with the same scaling behavior found in the discontinuous MCT transition and Random Field Ising model in a field. The dynamical transition is characterized by a static mixed order transition, in the same universality class of bootstrap percolation. This static transition is characterized by static critical fluctuations diverging only in the glassy phase, being absent in the liquid phase. Nevertheless the dynamics even in the liquid phase is strongly influenced by this static transition, as shown by the behavior of the dynamical heterogeneities, characterized by *χ*_4_(*t*). The presence of the static transition, at least at mean field level, characterized by a diverging static length, is responsible for the scaling laws and universality present in a wide range of dynamical critical phenomena, and sets the value of the upper critical dimensionality *d*_*c*_ = 8. In this scenario the sol-gel transition, which in mean field has been shown to be described by the continuous MCT model A^5^,^6^, can be considered as dynamical transition in a different universality class, characterized by the static random percolation transition with upper critical dimensionality *d*_*c*_ = 6.

## Methods

We performed Monte Carlo simulations of the FA kinetic facilitated model on a Bethe lattice. The Bethe lattice is a lattice extracted randomly from the set of lattices where each site is connected to *z* = *k* + 1 other sites. We consider a random lattice with *N* = 2^18^ sites, fixed coordination number *z* = *k* + 1 = 4, and *f* = 2. For each temperature we extract 32 different random lattices and initial configurations, and we start from a random configuration of the spins with density *p* = (1 + *e*^−1/*T*^)^−1^ of the up spins. Each Monte Carlo step is given by *N* spin flip trials. A spin flip trial consists in taking a random spin, and flipping it if it has *f* or more neighboring down spins, and with probabilities given by 
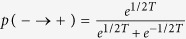
 and 
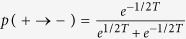
. For *k* = 3 and *f* = 2 the critical temperature is 
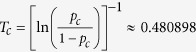
, with *p*_c_ = 8/9 and a fraction of blocked spins equal to *m*_c_ ≈ 0.673.

The relaxation function is defined as


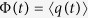


where 
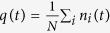
, and *n*_*i*_(*t*) = 0, 1 depending whether a spin at site *i* has flipped or not during time interval (0, *t*), while the fluctuations are defined as


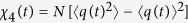


where 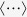 is the average over the thermal noise, the initial configurations and the random lattice.

## Additional Information

**How to cite this article**: de Candia, A. *et al.* Scaling and universality in glass transition. *Sci. Rep.* **6**, 26481; doi: 10.1038/srep26481 (2016).

## Supplementary Material

Supplementary Information

## Figures and Tables

**Table 1:** Numerical exponents obtained for the FA model on the Bethe lattice, the values of *c* and *y* are consistent with the scaling obtained from the cluster approach Eqs (12) and (13), the values of *z*, *a* and *b* are consistent with the MCT scaling relations Eqs (18) and (20).

z	a	b	c	y
2.7 ± 0.1	0.29 ± 0.01	0.50 ± 0.02	0.39 ± 0.01	0.46 ± 0.01
