# Supplementary material for: Scaling and universality in glass transition
Source: Sci Rep. 2016 May 25;6:26481. doi: 10.1038/srep26481 (PMC4879566; doi:10.1038/srep26481)
Supplement: Supplementary Information [file srep26481-s1.pdf]

Supplementary Information
Scaling and universality in glass transition

Antonio de Candia, Annalisa Fierro, Antonio Coniglio

March 1, 2016

The bootstrap percolation (BP) model [1] is defined in the following way. First, each site of the lattice is occupied by a particle randomly with probability p. Then, each particle that has less than m occupied neighbors (f or more empty neighbors, with $f = k + 1 - m$, and $k + 1$ being the coordination number) is removed, until a stable configuration is reached. The clusters of particles in this stable configurations are called m-clusters. On the Bethe lattice, which we will consider here, when $m > 2$ the model has a discontinuous transition. In this case there is a probability p_c, below which no particles are left at the end of the procedure, while for $p > p_c$, an infinite m-cluster appears with a finite density P, that can be considered the order parameter of the model. The subset of the m-cluster, formed by all the sites having exactly m neighbors, is called the "corona", while the sites having more than m neighbors are called the "deep core".

Bootstrap percolation has a mixed order transition: while the percolation order parameter P of BP jumps discontinuously at the threshold from zero to P_c, the fluctuation χ of the order parameter with respect to the initial configuration and the associated length ξ have a critical behavior given by $P - P_c \sim \epsilon^{\beta}$, $\chi \sim \epsilon^{-\gamma}$ and $\xi \sim \epsilon^{-\nu}$ where $\epsilon = |p - p_c|/p_c$

Here we show that, on the Bethe lattice, the critical exponent that characterizes the divergence of the fluctuations of the order parameter is $\gamma = 1$ and $\nu = 1/4$. For convenience, we also calculate the critical behavior of the order parameter P with critical exponent $\beta = 1/2$, the density of sites in the corona, and the divergence of the corona mean cluster size with an exponent $\gamma' = 1/2$.

The Bethe lattice is a lattice extracted randomly from the set of lattices where each site is connected to $z = k + 1$ other sites. Let us consider a Bethe lattice where each site is occupied with probability p. Define Q as the probability that a site, that has one of its neighbors belonging to the m-cluster, belongs itself to the m-cluster. By recursion, it has to satisfy the self-consistent equation $Q = pF(Q)$, where

$$F(Q) = \sum_{l=m-1}^{k} \binom{k}{l} Q^l (1-Q)^{k-l}. \quad (1)$$

When the occupation probability is greater than a threshold value p_c, a solution $Q > 0$ appears, that signals the presence of a m-cluster in the system. For $m \geq 3$, the function $F(Q)$ is a polynomial in Q with lowest order Q^2, and therefore the transition cannot be continuous. When the occupation probability reaches the critical value p_c, the probability Q jumps to a value $Q_c > 0$. Expanding $F(Q)$ in a Taylor series around Q_c, and taking into account that $F(Q_c) = Q_c/p_c$ and $F'(Q_c) = 1/p_c$, one finds, for $p \to p_c^+$,

$$Q - Q_c \sim |p - p_c|^{1/2}.$$

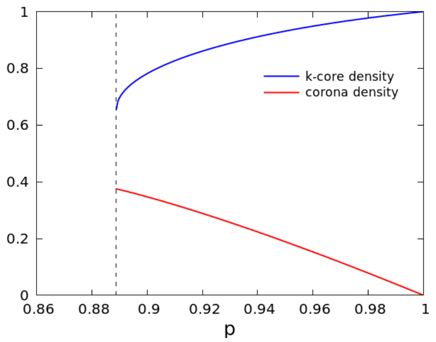

Figure 1: Density of the m-cluster and of the corona for $k = 3$ and $m = 3$. The critical point is $p_c = 8/9$.

The density P of the m-cluster and the density C of the corona can be expressed as

$$P = p \sum_{l=m}^{k+1} \binom{k+1}{l} Q^l (1-Q)^{k+1-l},$$

$$C = p \binom{k+1}{m} Q^m (1-Q)^{k+1-m}.$$

These quantities have a singularity at the critical point analogous to the one of Q. They jump to a finite value at $p = p_c$, and then they have a $|p - p_c|^{1/2}$ singularity for $p \to p_c^+$ (except for the case $k = 3$, $m = 3$, where the density of the corona is linear near the critical point). In Fig. 1 we show these densities in the case $k = 3$, $m = 3$.

Now consider two sites i and j having "chemical distance" n. The chemical distance is just the number of steps needed to go from one site to the other, given that on the Bethe lattice in the thermodynamic limit there is only one path joining the two sites. For a finite size lattice we are neglecting the presence of loops, and therefore multiple paths joining the sites, which is justified for $n \ll \log(N)$, where N is the total number of sites. In order to evaluate the pair correlation function $g_{i,j}$ and the fluctuations of the order parameter χ, let us first introduce lattice gas variables for the particles of the infinite cluster:

$$\pi_i = \begin{cases} 1 & \text{if } i \text{ belongs to the infinite } m\text{-cluster}, \\ 0 & \text{otherwise.} \end{cases}$$

It follows that for a lattice of size N,

$$P = \frac{1}{N} \sum_{i=1}^{N} \langle \pi_i \rangle \tag{2a}$$

$$g_{i,j} = \langle \pi_i \pi_j \rangle - \langle \pi_i \rangle \langle \pi_j \rangle \tag{2b}$$

$$\chi = \frac{1}{N} \sum_{i,j} g_{i,j} \sim \sum_n k^n g(n) \tag{2c}$$

where $g(n) = g_{i,j}$ with i and j having chemical distance n, and in the last step we took the thermodynamic limit, so that the number of sites at chemical distance n from a given site is proportional to k^n.

We want now to compute $\langle \pi_i \pi_j \rangle$, that is the probability that both i and j, having chemical distance n, belong to the infinite m-cluster. In Fig. 2 we show all the possible configurations of sites i and j, and of the $n-1$ sites between them. The

$$\text{a)}\quad p_0\ \overbrace{x\ \cdots\ x}^{(n-1)\ \text{times}}\ p_0$$

$$\text{b)}\quad p_0\ \theta\ \cdots\ \theta\ p_1$$

$$\text{c)}\quad p_1\ \theta\ \cdots\ \theta\ p_1$$

$$\text{d)}\quad p_0\ \overbrace{x\ \cdots\ x}^{s\ \text{times}}\ \theta_1\ \overbrace{\theta\ \cdots\ \theta}^{(n-2-s)\ \text{times}}\ p_1$$

$$\text{e)}\quad p_1\ \theta\ \cdots\ \theta\ \theta_1\ \theta\ \cdots\ \theta\ p_1$$

$$\text{f)}\quad p_1\ \overbrace{\theta\ \cdots\ \theta}^{r\ \text{times}}\ \theta_1\ \overbrace{x\ \cdots\ x}^{(n-3-s)\ \text{times}}\ \theta_1\ \overbrace{\theta\ \cdots\ \theta}^{(s-r)\ \text{times}}\ p_1$$

Figure 2: Possible configurations of the chain of sites from i to j, having chemical distance n (see text).

symbols p_0 and p_1 represent the possible states of sites i and j. p_0 (p_1) means that the site belongs to the m-cluster, has m or more (exactly $m-1$) neighbors belonging to the m-cluster among the k neighbors not shown in figure, (given that the neighbor shown in figure belongs to the m-cluster). The relative probabilities are

$$P_0 = p \sum_{l=m}^{k} \binom{k}{l} Q^l (1-Q)^{k-l},$$

$$P_1 = p \binom{k}{m-1} Q^{m-1} (1-Q)^{k-m+1}.$$

On the other hand, symbols x, θ and θ_1 represent the possible states of the $n-1$ sites between i and j. θ_1 (θ) means that the site belongs to the m-cluster, has $m-1$ or more (exactly $m-2$) neighbors belonging to the m-cluster among the $k-1$ neighbors not shown in figure, given that at least one (both) the neighbors shown in figure belong to the m-cluster. The relative probabilities are

$$\Theta_1 = p \sum_{l=m-1}^{k-1} \binom{k-1}{l} Q^l (1-Q)^{k-1-l},$$

$$\Theta = p \binom{k-1}{m-2} Q^{m-2} (1-Q)^{k+1-m}.$$

Note that $\Theta = \frac{p}{k} F'(Q)$, and therefore at the critical point Θ jumps to the value $\Theta_c = 1/k$. For $p \to p_c^+$ it has the same singularity as Q, therefore

$$1 - k\Theta \sim |p - p_c|^{1/2}.$$

Finally, x means that the site can be in any state, and the relative probability is one.

The probability $\langle \pi_i \pi_j \rangle$ is given by the sum of the six probabilities of the configurations of Fig. 2, that are given by

$$
\begin{aligned}
P_a &= P_0^2, \\
P_b &= 2P_0P_1\Theta^{n-1}, \\
P_c &= P_1^2\Theta^{n-1}, \\
P_d &= 2P_0P_1\Theta_1\sum_{k=0}^{n-2}\Theta^k = 2P_0P_1\Theta_1\frac{1-\Theta^{n-1}}{1-\Theta}, \\
P_e &= (n-1)P_1^2\Theta_1\Theta^{n-2}, \\
P_f &= P_1^2\Theta_1^2\sum_{s=0}^{n-3}(s+1)\Theta^k = P_1^2\Theta_1^2\left\{\frac{1-\Theta^{n-1}}{(1-\Theta)^2} - (n-1)\frac{\Theta^{n-2}}{1-\Theta}\right\}.
\end{aligned}
$$

The sum of all the terms of $\langle \pi_i \pi_j \rangle$ that do not depend on n gives $\left(P_0 + \frac{P_1\Theta_1}{1-\Theta}\right)^2$, that is equal to P^2. This can be verified using the relations

$$
\begin{aligned}
P &= P_0 + P_1 Q, \\
Q &= \Theta_1 + \Theta Q.
\end{aligned}
$$

In the first relation the probability that a site with $k+1$ neighbors belongs to the m-cluster P is given by the probability P_0 that at least m of the first k neighbors belong to it, plus the probability $P_1 Q$ that $m-1$ among the first k and the $(k+1)$-th neighbor belong to it. In a similar way the second relation holds for the probability Q that a site belongs to the m-cluster under the condition that the first neighbor also belongs to it. In conclusion, the terms that do not depend on n cancel out with the term $\langle \pi_i \rangle \langle \pi_j \rangle$ in Eq. (2b). Collecting all the other terms, we have

$$g(n) = (A + nB)\Theta^n,$$

where A and B go to a constant finite value for $p \to p_c^+$. As a consequence at leading order

$$\chi = \sum_n k^n g(n) \sim \sum_n n(k\Theta)^n \sim \frac{1}{(1-k\Theta)^2} \sim |p - p_c|^{-1},$$

so that the exponent that describes the fluctuations of the order parameter is $\gamma = 1$.

To evaluate the mean cluster size of corona clusters, we have to consider the corona connectivity $\Gamma(n)$, defined as the probability that two sites at a chemical distance n belong to the same corona cluster. In this case, the only contributing configurations are the ones of type c) in Fig. 2. Therefore the connectivity is $\Gamma(n) = P_1^2\Theta^{n-1}$, and the mean cluster size of corona clusters will be given by

$$\chi_c = \sum_n k^n \Gamma(n) \sim \sum_n (k\Theta)^n = \frac{1}{1-k\Theta} \sim |p - p_c|^{-1/2},$$

so that the critical exponent is $\gamma' = 1/2$, in agreement with Ref. [2] and [3].

Finally, we consider the exponent ν, that describes how the correlation length ξ diverges approaching the critical point. It has to be considered that the chemical distance n on the Bethe lattice plays the role of the square of the euclidean distance r^2, because the lattice is infinite dimensional. We can then define a pair correlation function using the distance r:

$$\frac{f(r/\xi)}{r^{d-2+\eta}} r^{d-1} dr \sim g(n) k^n dn.$$

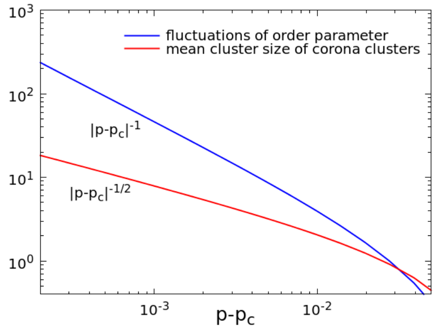

Figure 3: Mean cluster size of corona clusters and fluctuations of the order parameter as a function of $p - p_c$.

Being $g(n) = n\Theta^n$, we have

$$\frac{f(r/\xi)}{r^{\eta-1}}dr = r^2 (k\Theta)^{r^2} rdr = r^3 f(r/\xi)dr$$

where

$$\xi = \sqrt{(\ln k\Theta)^{-1}} \sim |p - p_c|^{-1/4},$$

showing that $\nu = 1/4$ and $\eta = -2$. In a similar way for the connectivity of the corona cluster we find the same exponent $\nu = 1/4$ for the correlation length and $\eta = 0$, in agreement with the results of Ref. [3].

References

[1] Chalupa, J., Leath, P. L., & Reich, R. Bootstrap percolation on a Bethe lattice, *J. Phys. C: Solid State Phys.* **12**, L31 (1979).

[2] Goltsev, A.V., Dorogovtsev, S.N. & Mendes, J.F.F. k-core (bootstrap) percolation on complex networks: Critical phenomena and nonlocal effects, *Phys. Rev. E* **73**, 056101 (2006).

[3] Schwarz, J. M., Liu, A. J., & Chayes, L.Q. The onset of jamming as the sudden emergence of an infinite k-core cluster, *Europhys. Lett.* **73**, 560 (2006).
